# Absorption of Energy in Excess, Photoinhibition, Transpiration, and Foliar Heat Emission Feedback Loops During Global Warming

**DOI:** 10.3390/cells15010075

**Published:** 2026-01-01

**Authors:** Roshanak Zarrin Ghalami, Maria Duszyn, Stanisław Karpiński

**Affiliations:** Department of Plant Genetics, Breeding, and Biotechnology, Institute of Biology, Warsaw University of Life Sciences, 02-776 Warsaw, Poland; roshanak_zarrin_ghalami@sggw.edu.pl (R.Z.G.); maria_duszyn@sggw.edu.pl (M.D.)

**Keywords:** global warming, excess energy absorption (AEE), photoinhibition, non-photochemical quenching (NPQ), foliar heat emission, reactive oxygen species (ROS), transpiration, terrestrial photosynthesis

## Abstract

**Highlights:**

**What are the main findings?**

- Foliar temperature response during excess light stress is the result of a dynamic interplay between NPQ, qE, photochemistry, and transpiration rather than of any single mechanism. Thus, spatial and temporal heterogeneity of leaf temperature arises from the physiological state and absorbed energy fate balance.
- Conditionally, the ratio between these processes can vary, thus increasing or decreasing foliar heat emission and warming up or cooling down the surrounding environment.
- Constraints imposed by global warming and drought can further disrupt this balance, increasing the risk of foliar heat stress and cell death induction.

**What are the implications of the main finding?**

- Leaf-internal energy redistribution represents an important but often simplified and ignored component of foliar thermoregulation under excess light and climate warming.
- Explicit consideration of these interactions may improve understanding and modelling of plant–climate feedbacks under future warming scenarios.

**Abstract:**

Global warming is increasingly constraining plant productivity by altering the photosynthetic energy balance and leaf thermoregulation. Under high light and elevated temperatures, absorption of energy in excess (AEE) by photosystem II disrupts photosynthetic electron transport, oxygen evolution, and CO_2_ assimilation, often accompanied by reduced foliar transpiration. These conditions promote photoinhibition, as reflected by a decrease in maximal photosynthetic efficiency (Fv/Fm), an increase in non-photochemical quenching (NPQ), and photooxidative stress associated with enhanced reactive oxygen species (ROS) production. In addition to environmental heat stress, AEE influences foliar temperature through internal energy partitioning, including regulated dissipation of AEE as heat and changes in transpirational cooling. The relative contributions of NPQ, photochemistry, and transpiration to leaf temperature regulation are strongly context dependent and vary with light intensity, temperature changes, and water availability. Under global warming, rising background temperatures and increased vapor pressure deficit may constrain transpirational cooling and alter the balance between non-photochemical and photochemical energy dissipation and usage, respectively. In this review, we synthesize current knowledge on AEE handling, photoinhibition, NPQ and other quenching processes, and on transpiration cooling, and discuss a conceptual framework in which sustained imbalance among these processes under global warming conditions could amplify foliar heat stress and increase the risk of cellular damage. Rather than proposing new physiological mechanisms, this work integrates existing evidence across molecular, leaf, and ecosystem scales to highlight potential feedbacks relevant to plant performance under future climate prediction scenarios.

## 1. Introduction

### Global Warming Impacts on Environmental Stability

The complex link between plant stress responses and global warming has far-reaching implications for environmental stability and food security. Plants serve as important carbon sinks, but rising temperatures and heat stress weaken their ability to absorb carbon dioxide (CO_2_), accelerating climate change by increasing the buildup of greenhouse gases in the atmosphere, escalating the greenhouse effect [1,2,3,4,5]. This cycle intensifies global warming, threatening biodiversity and food security, especially in vulnerable regions such as dry and semi-arid zones [6]. Moreover, climate change reduces agricultural productivity, as crucial crops such as rice, wheat, and maize are affected by heat stress, resulting in lower yields and reduced quality [3]. In cereal species such as wheat and barley, combined heat and drought stress severely impacts grain development, reducing both kernel number and weight, as well as overall productivity [7,8,9,10]. Given these challenges, improving the thermal resilience of crops through breeding programs, irrigation optimization, and precision agriculture is crucial for sustaining food production in the face of climate change. Furthermore, the pressure to expand farmland into forests and wetlands, driven by the need to increase agricultural production, further accelerates deforestation, resulting in increased greenhouse gas emissions and biodiversity loss [11].

Wildfires represent a critical interface between plant physiological stress responses, ecosystem function, and global carbon cycling [12,13]. Large-scale fire events rapidly release carbon dioxide through the combustion of aboveground biomass and soil organic matter, while simultaneously reducing long-term carbon sequestration capacity by increasing tree mortality, degrading soil structure, and simplifying canopy architecture [14,15]. Although intact forests function as major terrestrial carbon sinks through sustained photosynthetic carbon assimilation, repeated high-severity fires can shift ecosystems from carbon sinks to persistent carbon sources, thereby amplifying atmospheric CO_2_ accumulation and reinforcing positive climate feedback loops [16,17]. Climate change-induced wildfires, driven by prolonged droughts and extreme heatwaves, have become more frequent and severe, further accelerating deforestation [18]. In 2019 alone, the Amazon rainforest experienced substantial forest loss, with over 906,000 hectares lost to fires [19,20]. This extensive biomass combustion released significant amounts of carbon dioxide into the atmosphere, thereby reducing the forest’s capacity to sequester carbon and weakening its role in regulating the global climate [21,22]. In 2018, Sweden’s fires destroyed 25,000 hectares of forest, resulting in extensive ecological devastation, financial loss for the wood sector, and the evacuation of several villages [23]. Similar wildfire events in recent years have devastated landscapes in California, Australia, and Russia’s taiga, causing ecological and economic losses [24,25,26]. Deforestation caused by these fires, combined with the ongoing kinetic war in Ukraine since 2022, releases unprecedented amounts of carbon dioxide into the atmosphere, further accelerating climate change through an autocatalytic process [27,28]. Beyond direct carbon emissions, fire-induced deforestation and canopy loss strongly modify local and regional microclimates [29]. Reduced canopy cover decreases shading and transpiration-driven evaporative cooling, leading to higher foliar and air temperatures in post-fire environments. These changes exacerbate heat stress in regenerating vegetation, impair photosynthesis, and further constrain carbon uptake during recovery phases [30,31]. Consequently, wildfire impacts extend beyond immediate biomass loss, influencing long-term plant thermal environments and physiological performance. It is well known that forests play a crucial role in maintaining soil fertility and hydrological cycles [11,25]. When deforestation occurs, soil quality degrades, making agricultural land less productive and more prone to erosion, which in turn rapidly reduces its capacity to sustain long-term agricultural output [11]. Additionally, tree loss disrupts regional rainfall patterns, leading to prolonged droughts, reduced water availability, and greater crop vulnerability to pests [32]. It also diminishes soil fertility, increasing the risk of nutrient depletion and landslides in agricultural regions. Once forest cover is removed, nutrient-poor soil quickly loses its ability to support long-term cultivation [11,13]. This unsustainable cycle ultimately threatens food security by degrading soil quality, depleting water resources, and heightening agricultural vulnerability [28]. The interplay between deforestation, agricultural expansion, and climate change reinforces a feedback loop in which land degradation and declining productivity further drive deforestation and carbon emissions [33]. From a plant-physiological perspective, these land-use changes intensify thermal stress at the leaf level by increasing background temperatures, reducing canopy humidity, and limiting transpirational cooling [34]. At the level of individual leaves, independent evidence further indicates that thermal heterogeneity can arise from internal energy partitioning rather than from uniform environmental heating. Kulasek et al. (2016) demonstrated that temperature differences between chlorophyll-rich (green) and chlorophyll-poor (cream) sectors within the same leaf originate primarily from differences in absorbed excitation energy and its dissipation as heat within chloroplast-containing tissues. Because these contrasting sectors experience identical external conditions, this finding underscores that foliar temperature patterns can be shaped by where and how absorbed radiation is dissipated within the leaf, rather than solely by background temperature. Together with our observations of spatially heterogeneous foliar warming, these results support the view that plant thermal responses to intensified environmental stress reflect both ecosystem-level drivers (e.g., deforestation-induced warming and reduced humidity) and leaf-internal energy redistribution mechanisms [35,36].

The elevated Foliar temperatures predispose plants to photoinhibition, oxidative stress, and reduced carbon assimilation, directly linking ecosystem-scale disturbances to cellular heat stress responses [37]. The impact of global warming on forests and agriculture is therefore profound. Heat stress impairs plant resilience, reducing crop productivity and food security. Therefore, understanding temperature stress and plant responses is crucial for future food security and sustaining the quality of life. In contrast, prescribed and low-intensity fires can contribute to climate mitigation when applied within ecologically appropriate fire regimes [38]. By reducing fuel loads and preventing large, uncontrolled wildfires, such fires help preserve mature trees, maintain soil integrity, and promote forest regeneration dominated by younger, rapidly growing cohorts with high carbon uptake potential [38]. Controlled burning can also enhance belowground carbon stabilization through the formation of recalcitrant charcoal and stimulation of root turnover, contributing to longer-term soil carbon storage [17]. Collectively, fire regimes influence not only ecosystem-scale carbon dynamics but also plant thermal environments by shaping canopy structure, transpiration capacity, and foliar heat balance, thereby linking wildfire activity to plant thermoregulation and global warming feedbacks.

The theoretical contribution of this review lies in integrating established photoprotective and thermoregulatory processes into a coherent conceptual framework. While individual mechanisms such as NPQ, photoinhibition, and transpiration have been extensively studied, their combined influence on foliar thermal balance under global warming has received less explicit attention. By linking excess excitation energy handling with spatial foliar temperature dynamics and environmental constraints on transpirational cooling, this review provides a cross-scale perspective that complements existing molecular and ecosystem-focused studies.

## 2. Thermoregulation in Plants

Recently, Griffani et al. (2024) discussed how foliar temperature can differ from ambient temperature, highlighting the role of thermodiffusion in driving transpiration and presented a new model that incorporates thermodiffusion, showing how temperature gradients between the leaf and air influence transpiration, with particular focus on conditions that affect reverse transpiration and the impact of thermal imbalance [39]. Another study quantitatively assessed the role of non-photochemical quenching (NPQ) in dissipating excess energy as heat, estimating its contribution at 63.9 W m^−2^ when exposed to 888 µmol m^−2^ s^−1^ PAR sunlight. A thermodynamic leaf model incorporated thermal conductivity and the impact of NPQ on foliar temperature, while thermal imaging confirmed the correlation between foliar temperature and PSII efficiency [40]. Murakami et al. (2024) theoretically quantified NPQ heat production at 63.9 W m^−2^ under direct sunlight (376 W m^−2^ PAR), representing ~30% of total foliar heat emission (~206 W m^−2^), with the remaining ~70% arising from constitutive PSII/PSI electron transport inefficiencies and respiration independent of NPQ regulation (pH, xanthophylls, PsbS). While this work quantified the NPQ-dependent component of AEE dissipation as heat theoretically, the present review extends that framework by considering experimental evidence on how NPQ interacts with photochemistry and transpiration to determine the net foliar temperature response under AEE conditions. Crucially, NPQ constitutes the primary regulated mechanism controlling AEE dissipation as heat under fluctuating high light and stress, distinguishing it from unavoidable baseline fluxes. The recent review by Bernacchi et al. (2025) outlines biochemical and physiological mechanisms by which rising temperatures impair photosynthesis in crops, highlighting enzyme thermolability, photorespiration, and canopy traits as targets for improving heat resilience through breeding and biotechnology [41]. The authors present promising genetic and metabolic strategies to enhance photosynthetic thermotolerance and sustain yields in a warming climate. Mechanistic models complement this view by demonstrating that enhancing Rubisco activase thermostability and modifying photorespiration can additively improve photosynthetic carbon assimilation at temperatures above the thermal optimum, consistent with empirical data from transgenic plants [42]. Nevertheless, understanding the dynamic regulation of foliar temperature through mechanisms such as transpiration and NPQ remains crucial, as foliar temperature can differ significantly from ambient temperature due to these physiological processes. Reduced transpirational cooling has long been recognized in plant physiological studies as a primary driver of elevated leaf temperature [43]. However, classical energy balance frameworks typically treat leaf warming primarily as a consequence of reduced evaporative cooling and do not resolve the internal partitioning of absorbed energy within the photosynthetic apparatus. Recent studies emphasize the significance of understanding whole-ecosystem energy fluxes, including terawatt-scale AEE dissipation as heat by vegetation, which is crucial for accurate modeling of photosynthetic heat stress and thermoregulation [36]. Field observations further suggest that long-term environmental drivers can modulate these processes at the canopy scale. Elevated atmospheric CO_2_ has been shown to increase canopy temperatures by reducing transpiration cooling, indicating that physiological regulation of stomata can indirectly influence canopy heat balance [44]. Moreover, considering the impacts of heat stress beyond the leaf scale, including canopy architecture and ecosystem microclimates, is essential for a comprehensive understanding of photosynthetic performance under elevated temperatures. However, recent work has shown that canopy-scale temperature measurements can substantially underestimate the thermal stress experienced by individual sun-exposed leaves [45]. demonstrated that reliance on canopy temperature leads to strong overestimation of leaf thermal safety margins, as the most exposed leaves can approach photosynthetic tolerance limits despite moderate canopy temperatures. Integrating these multiscale physiological and environmental factors will be key to developing crops with improved resilience in the face of climate change. Some plants possess a unique ability to regulate temperature, enabling them to adapt to various environmental conditions. Notably, thermogenic plants such as the sacred lotus (*Nelumbo nucifera*) and eastern skunk cabbage (*Symplocarpus foetidus*) can tolerate elevated temperatures, which enhances their reproductive success. Plants are continuously exposed to fluctuating temperatures and light intensities, requiring effective thermoregulatory mechanisms to optimize photosynthesis, respiration, and growth versus stress adaptation and cell death induction [46,47,48,49,50]. One of the most thermally sensitive components of the photosynthetic apparatus is photosystem II (PSII). The primary site of heat-induced dysfunction within PSII is the oxygen-evolving complex (OEC), which is partially ligated by the D1 protein and is particularly vulnerable to elevated temperatures. Heat stress impairs oxygen evolution by destabilizing the OEC, thereby compromising PSII function and predisposing the reaction center to subsequent photodamage. Under high-light conditions, excess excitation energy promotes the formation of reactive oxygen species, which further exacerbate PSII photoinhibition [51]. As a consequence of OEC destabilization and oxidative stress, the D1 protein an essential core component of the PSII reaction center involved in charge separation becomes damaged and must be replaced [52,53]. Damaged D1 is rapidly recognized, removed by thylakoid proteases, and replaced by newly synthesized protein, enabling PSII to recover activity even under adverse conditions [52,53]. The rate of D1 turnover is dynamically regulated in response to light intensity, PSII phosphorylation status, and the redox state of the thylakoid membrane, ensuring that repair processes keep pace with damage. Through this tightly controlled repair cycle, D1 renewal contributes to the maintenance of PSII functionality and photosynthetic efficiency under fluctuating high-light and high-temperature environments, while the integrity of the OEC remains a critical determinant of thermal sensitivity [51,52,53].

## 3. Non-Photochemical Quenching and Foliar Temperature Regulation

**Figure 1:**
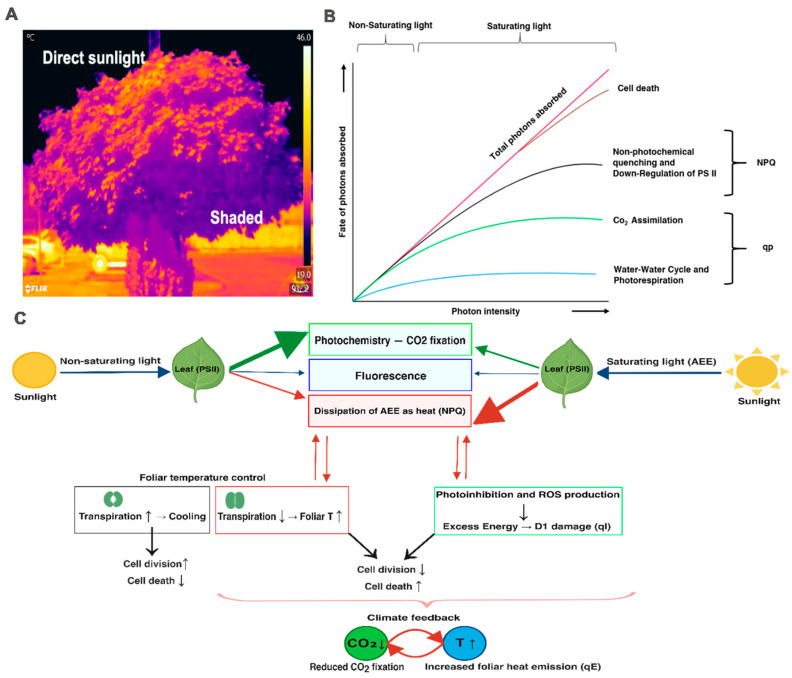
Fate of absorbed light energy in leaves and its role in global warming feedbacks. (**A**) Representative thermal image of *Acer platanoides* leaves under field conditions, showing higher foliar temperature in sunlight-exposed regions compared with shaded areas. This panel illustrates spatial differences in leaf temperature arising from contrasting irradiance and transpirational cooling, but does not directly resolve underlying photochemical processes. (**B**) Conceptual overview of energy partitioning under increasing light intensity. Under non-saturating light and optimal stomatal conductance, most of the absorbed energy is utilized in photochemistry. Under saturating light, absorbed energy in excess (AEE) accumulates and must be dissipated through a combination of regulated (e.g., NPQ) and unregulated pathways, particularly when transpirational cooling is constrained (Adapted from Asada, 1999) [56] (**C**) Schematic representation of the main fates of excitation energy absorbed by chlorophyll (blue arrow), partitioned among (i) photochemistry and charge separation (green arrow), (ii) chlorophyll fluorescence (blue arrow), and (iii) AEE dissipation as heat (red arrow) via NPQ and other mechanisms. Under optimal conditions, open stomata support transpiration-driven cooling and CO_2_ uptake, maintaining lower foliar temperatures. Under AEE, drought or heat stress reduces stomatal closure, which in turn increases foliar temperature and enhances the relative importance of heat dissipation pathways. NPQ contribution in this figure is not inferred directly from thermal imaging, but is indicated based on established photophysiological knowledge and published studies [57,58]. Reduced CO_2_ uptake, together with enhanced heat emission and ROS production, may establish a positive feedback framework in which elevated temperature promotes stomatal closure and increased dissipation of excess energy as heat via NPQ. Under prolonged stress, such conditions have been associated with ROS-mediated cellular damage and programmed cell death in leaves [68]. At larger spatial scales, the cumulative effects of these processes have been proposed to influence ecosystem energy balance and climate feedbacks.

Research on foliar temperature, its regulation, and perception and response to external heat and high-intensity light is explored and analyzed across various levels and contexts, including molecular and physiological aspects. Most studies on NPQ primarily focus on its molecular and physiological mechanisms, often neglecting its global impact on foliar heat emission and the quantification of this process [54,55]. Plants possess a natural capacity to absorb energy in excess (AEE) of what is sufficient for photosynthetic electron transport and photochemistry; thus, in full sunlight, only a fraction of light absorbed by chlorophylls in light-harvesting complexes (LHC) is utilized for CO_2_ fixation. In essence, plants convert absorbed light into three energy channels: fluorescence, heat, and photochemistry (Figure 1A) [54,56]. Under optimal and non-saturating light conditions, the vast majority of the energy is used for photochemistry. However, when the sun is high, AEE must be dissipated as heat by the NPQ mechanism [35,57,58,59]. This heat is generated primarily inside the chloroplasts by PSII during the conversion of AEE into heat. While NPQ and other energy quenching components represent a regulated and dynamically adjustable system of AEE dissipation as heat, it is important to note that a substantial fraction of absorbed light energy is inevitably released as heat through photochemical inefficiencies, charge recombination, and downstream metabolic processes in PSII and PSI, independent of NPQ regulation. Beyond dissipation of AEE as heat by NPQ and transpiration, which serves as a primary cooling mechanism [60], plants employ additional protective strategies to regulate AEE and prevent photodamage. These include, e.g., cyclic electron flow, alternative electron transport pathways, water-water cycle, photoinhibition, and downregulation of PSII [56,57,61,62], and chlorophyll-to-carotenoid energy transfer [63], which facilitates energy dissipation and heat regulation on ultrafast timescales (Figure 1B). When drought reduces transpiration rate, thus foliar cooling, stomatal closure can raise foliar temperature by ~2 °C [36], making NPQ-driven AEE dissipation as heat a key mechanism for regulating foliar temperature, particularly in moderate air temperatures and high-light environments. During the 2018 European heatwave, Martini et al. (2022) observed NPQ saturation in Quercus ilex under 43 °C, inverting the photochemistry-fluorescence relationship as energy shifted from qE/qI protection toward sustained photoinhibition, elevating foliar heat load when transpiration cooling failed, highlighting AEE dissipation limits that exacerbate warming feedbacks [64]. However, Antala et al. (2025) suggest that stomatal limitation by high vapor pressure deficit primarily drove the decoupling of photosynthesis from chlorophyll fluorescence, rather than NPQ exhaustion, emphasizing the integrated NPQ-stomatal interactions in foliar thermoregulation under extreme AEE [65,66]. At the ecosystem level, the coordination between transpiration (cooling), photoinhibition, and NPQ is essential for maintaining foliar temperature homeostasis. Under conditions of water limitation, when stomatal closure restricts transpiration cooling, photoinhibition of the photosynthetic electron transport at PSII and NPQ becomes increasingly significant as a mechanism to restrict absorption AEE or increase AEE dissipation as heat [40]. Interestingly, chemical inhibition of photosynthetic electron transport, such as through 3-(3,4-dichlorophenyl)-1,1-dimethylurea (DCMU) treatment, has been shown to reduce foliar temperature despite lowering photochemical efficiency.

This seemingly paradoxical effect suggests that photoinhibition, in some contexts, may actively contribute to thermal regulation by minimizing AEE input into heat-generating pathways (NPQ, qE, and others). Such findings highlight the existence of a finely tuned regulatory network in which charge separation and photosynthetic electron transport, non-photochemical processes (NPQ, qE, and others), and transpiration cooling dynamically interact to adjust foliar heat load balance in response to AEE and temperature stress [36]. This internal foliar heat generation and its regulation in chloroplasts due to AEE dissipation as heat was not discussed and considered in recently published reviews about temperature sensing and plant responses [42,67]. It is difficult to distinguish NPQ-dependent heat generation from passive heat from the environment; therefore, photo-calorimetric measurements performed under controlled light intensity can be applied to address this limitation. Recent measurements, using a custom-made photo-calorimeter under defined blue/red LED illumination (1980 μmol m^−2^ s^−1^) and a stable ambient temperature (~21–22 °C), illustrate how such an approach can be used to discuss the internal coordination between NPQ, photochemistry, and transpiration. These experimental observations strongly suggest that not only do external surroundings determine plants’ temperature responses, but also, internal homeostasis between non-photochemical and photochemical processes in chloroplasts during AEE is responsible for the final foliar heat load [36].

After exposure to high irradiance, NPQ remains active, dissipating the AEE as heat, even when the light intensity becomes limiting for photosynthesis. The observed delayed relaxation of NPQ reduces the efficiency of photon utilization for CO_2_ fixation in low light, resulting in a transient decrease in photosynthetic efficiency. This inefficiency highlights a critical limitation in photosynthetic performance under fluctuating light conditions, typical in natural environments [69]. NPQ prevents photodamage via two main components: the fast-relaxing qE and the slow-relaxing qI. Among these components, the rapidly reversible energy-dependent quenching (qE) constitutes the dominant regulated mechanism of thermal energy dissipation under excess light, whereas the slowly relaxing component reflects sustained quenching associated with photoinhibitory damage [70,71]. qE depends on PsbS-mediated antenna reorganization and zeaxanthin-enhanced energetic connectivity between PSII supercomplexes, which facilitates lateral energy transfer and efficient AEE dissipation as heat. The emergence of qI indicates that these protective mechanisms have been exceeded. Under high-light conditions (AEE), a substantial fraction of absorbed excitation energy is dissipated as heat via non-photochemical quenching, often becoming the dominant energy sink when photochemical capacity is exceeded [72]. Xanthophylls interconvert between zeaxanthin and violaxanthin in a pH-dependent manner, modulating qE. Proton and xanthophyll binding to antenna complexes induce conformational changes in PSII and PsbS. While qE reverses quickly, qI reflects slower quenching, mainly driven by photoinhibition [55,73,74]. PsbS acts as a pH sensor in the chloroplast lumen, triggering structural changes in the light-harvesting complex to release AEE as heat [75]. Arabidopsis *npq4-1* mutants, which lack PsbS and exhibit impaired NPQ, fail to regulate AEE effectively, resulting in photodamage [35,76]. However, the interplay between PsbS, NPQ, and AEE dissipation as heat remains a topic of debate. Although PsbS is a key protein in the induction of qE, the exact mechanism of its action remains unclear [35,77]. A simple model suggests that reduced photosynthetic efficiency, leading to higher NPQ and AEE dissipation as heat, results in increased foliar temperature. Experiments conducted on high-light-acclimated leaves, which exhibit enhanced NPQ, demonstrated a stronger light-induced heating effect [35,59]. It was concluded that there is a correlation between the extent of NPQ and the increase in light-induced foliar temperature [78]. NPQ influences stomatal behavior, with functional NPQ promoting stomatal opening to facilitate transpiration and CO_2_ uptake, whereas impaired NPQ results in stomatal closure, elevating foliar temperature and increasing the risk of photodamage [55,74]. Studies have shown that *npq4-1* mutants tend to undergo stomatal closure under AEE. This suggests that functional PsbS and NPQ are required for maintaining stomatal opening during AEE, facilitating transpiration and CO_2_ uptake, and that compromised NPQ may contribute to stomatal closure [35].

Studies on several species suggest that foliar temperature is a complexly regulated parameter influenced by both transpiration, which acts as a cooling mechanism, and photosynthetic processes, as evidenced by the reduction in foliar temperature upon generic inhibition of the photosynthetic electron transport and NPQ with DCMU [36]. NPQ does not necessarily increase net leaf heat load under all conditions but may instead redistribute absorbed excitation energy toward rapid dissipation as heat, thereby altering the spatial and temporal pattern of foliar heating. Taken together, these observations suggest [78] that in plants, mechanisms linked to absorbed energy dissipation, including NPQ, contribute to foliar thermoregulation under AEE conditions and underscore the intricate balance between photoprotection, gas exchange, water use efficiency, and energy efficiency in plants [78]. Thus, the balance between NPQ-mediated dissipation of AEE as heat, photosynthetic electron transport efficiency, and overall plant transpiration cooling remains an area of active research, with ongoing studies aimed at quantifying temperature variations across different plant species and environmental conditions [78]. These physiological interactions carry implications that extend far beyond the function of individual leaves. At the canopy scale, particularly in dense vegetative systems such as tropical forests, the cumulative dissipation of AEE as heat by NPQ and other energy quenching mechanisms can contribute substantially to energy flux and microclimate regulation. While NPQ operates at the chloroplast level, its cumulative effects across leaves and canopies may collectively influence ecosystem-scale energy fluxes. Consistent with this scaling complexity, recent analyses show that thermal optima for photosynthesis differ markedly between leaf and canopy levels. Kumarathunge et al. (2024) demonstrated that canopy-level photosynthetic temperature optima are substantially lower than leaf-level optima, reflecting the integration of shaded leaves, vertical gradients within the canopy, and heterogeneous energy dissipation [79]. While transpiration has long been considered the dominant form of vegetative cooling, the thermodynamic role of NPQ in buffering foliar heat flux remains underrepresented in current climate models. Given the increasing frequency of drought and heat stress, there is a growing recognition that NPQ should be explicitly integrated into land surface energy exchange frameworks. Incorporating this photoprotective mechanism could significantly improve our understanding of how vegetation modulates its thermal environment and, in turn, influences atmospheric dynamics [36,71]. It is essential to recognize that NPQ changes are not only induced by light absorption but also triggered by wounding or point burning, and they propagate systemically through electrical, phytohormonal, and ROS signaling [80,81,82]. Wave-like NPQ, ROS, and electrical signaling propagate across leaves and from leaf to leaf, and are essential for the induction of the systemic and the network acquired acclimation (SAA and NAA) [80,82,83]. NPQ changes and PsbS levels are also important for regulating cellular light memory, which is crucial for light acclimation responses [83]. Thus, PsbS-dependent NPQ changes are not only important in AEE dissipation as heat but are also important in the regulation of chloroplast local, systemic, and network retrograde acclimation signaling within and between leaves to AEE, UV, wounding, and heat stress responses, and in the regulation of stomatal conductance and foliar cell death [58,68,80,82,83,84]. NAA and SAA responses involve rapid systemic signaling through electrical signals, ROS, and NPQ waves that coordinate stress responses within and between plants. During these processes, NPQ is systemically modulated in a wave-like manner, facilitating enhanced photoprotection and thermoregulation across physically connected plant leaves, thereby preparing both local and distant leaves for impending environmental stress [58,85]. These signals induce dynamic adjustments in the distribution of absorbed energy at the photosystem II level, integrating molecular, physiological, and spatial responses that maintain photosynthetic efficiency and enhance acclimation under fluctuating light and temperature conditions. This raises the intriguing possibility that dynamic modulation of NPQ during NAA and SAA may indirectly affect foliar thermal status, although further experimental evidence is needed to confirm this link. This emerging understanding highlights plants’ capacity for integrated network-level coordination of photoprotection and thermoregulation within plant communities.

In addition to its role in light acclimation, NPQ changes and chloroplast retrograde signaling participate in broader physiological and cell death immune responses. The LESION SIMULATING DISEASE1 (LSD1) protein has been associated with NPQ regulation and cross-talk between light stress acclimation and immunological signaling, illustrating the complex regulatory networks that control plant responses to environmental stressors [68]. Moreover, it has been demonstrated that CRK5 regulates both NPQ and stomatal conductance, thereby influencing foliar temperature under excess light. Mutants lacking CRK5 exhibit reduced NPQ and chlorophyll content, resulting in lower foliar temperatures, which suggests a link between CRK5, NPQ, and stomatal function in response to light stress. Additionally, CRK5 is associated with a salicylic acid-related pathway, suggesting a potential role of plant hormones in regulating foliar temperature [86,87].

Similarly, MPK4, which is involved in regulating SA-dependent cell death and immune responses, also plays a role in controlling NPQ, stomatal conductance, and foliar thermoregulation. Field-grown transgenic poplar trees displayed smolder growth and higher NPQ and foliar temperature when stomata were open, and transpiration was higher [59]. In *Arabidopsis thaliana*, CIA2 and its homolog CIL regulate heat tolerance (HT) through transcriptional control of chloroplast function and stress signaling [88]. They act as negative regulators of thermotolerance by repressing heat shock response genes, and their inactivation enhances heat stress resilience. This suppression is linked to altered expression of nuclear-encoded chloroplast-related genes, influencing chloroplast-to-nucleus signaling under thermal stress. Double mutants (*cia2/cil*) show increased thermotolerance and elevated expression of heat-responsive genes such as *HSP70* and *HSP101* [88]. Beyond thermotolerance, CIA2 and CIL also affect chloroplast protein import and nuclear gene regulation [89]. Transcriptomic analyses further reveal that loss of CIA2/CIL upregulates stress-related pathways, including chaperone activity and ROS detoxification, processes vital for heat stress survival [88]. Thus, while central to chloroplast biogenesis, CIA2/CIL act as repressors of heat stress resilience, highlighting a trade-off between chloroplast functionality and thermotolerance [88,89]. These foliar temperature regulators were also not considered in recently published reviews about temperature sensing and plant responses [42,67].

Together, these examples illustrate how non-photochemical and photosynthesis-linked processes actively contribute to foliar temperature regulation, motivating a broader conceptual reassessment of plant energy balance. Previous reviews on plant responses to heat and high light have primarily focused on external temperature stress, photoinhibition, transpiration-driven cooling, and biochemical limitations of photosynthesis, often treating foliar temperature as a consequence of environmental temperature conditions [41,42,46,48]. In contrast, the present review emphasizes that AEE inherently leads to internal heat generation within chloroplasts as a direct outcome of photochemical and non-photochemical energy partitioning. Specifically, this review integrates (i) NPQ-dependent AEE dissipation as heat as an active and regulated process, rather than a passive by-product, (ii) the dynamic interplay between NPQ, photochemistry, and transpiration in shaping net foliar temperature responses, and (iii) spatial and temporal heterogeneity of leaf temperature arising from physiological state and energy balance. Conditionally, the ratio between these processes can increase or decrease foliar heat emission; thus, it can globally influence the environment. By shifting the focus from environmentally induced heat stress to internally generated foliar thermal loads driven by AEE, NPQ, and other energy quenching processes, and by transportational cooling limitations this review extends existing frameworks and highlights feedback mechanisms that are not captured in earlier syntheses. In this context, incorporating AEE-driven internal heat into existing views of leaf thermoregulation alters the interpretation of plant energy balance by recognizing absorbed energy partitioning (photon fate) as an active contributor to leaf temperature. Together, these considerations define a conceptual framework rather than a quantitative model, complementing and extending existing leaf energy balance and thermoregulation models by explicitly incorporating AEE-driven internal NPQ and other energy quenching processes leading to internal foliar heat generation and its interaction with photochemistry and transpiration.

## 4. Passive and Active Mechanisms of Plant Thermotolerance

Phytohormones constitute a central regulatory layer in plant thermotolerance [90]. However, as highlighted in recent integrative reviews, plant heat tolerance is not governed by a single pathway but emerges from a multi-layered hierarchy of mechanisms, beginning with passive heat avoidance traits, followed by active physiological regulation and longer-term transcriptional and metabolic reprogramming. These layers collectively determine how plants perceive heat stress, dissipate excess energy, and maintain cellular homeostasis under elevated temperatures [34,90]. In addition to active physiological processes such as transpiration and non-photochemical quenching (NPQ), plants rely on a suite of passive defense mechanisms that limit excessive foliar heat accumulation. These adaptations are primarily structural and morphological, functioning by reducing radiation absorption, enhancing convective and radiative heat loss, and minimizing thermal load without requiring direct metabolic energy investment [47,91]. As emphasized in the second review, such passive traits establish the baseline thermal environment of the leaf, upon which hormonal and molecular responses subsequently act. At the leaf level, reduced leaf size, increased leaf dissection, and narrower laminae decrease boundary layer thickness, facilitating more efficient convective heat exchange with the surrounding air and lowering Foliar temperature under high irradiance [91,92]. Alterations in leaf orientation, including paraheliotropic movements and steeper leaf angles, further reduce direct solar interception during peak radiation periods. Reflective surfaces formed by waxy cuticles, trichomes, or thick epidermal layers decrease absorptance of incoming radiation and promote radiative heat dissipation [93]. These traits are particularly prominent in species adapted to arid and semi-arid environments, where evaporative cooling is frequently constrained.

Passive defenses also operate at the canopy and whole-plant scale. Architectural complexity, self-shading, and spatial heterogeneity in leaf arrangement buffer internal Foliar temperatures and reduce thermal extremes during heat waves [91]. As discussed in the second review, these canopy-scale traits reduce thermal heterogeneity within plant tissues, thereby lessening the need for energetically costly protective responses. Although passive mechanisms alone cannot fully prevent overheating during extreme or prolonged heat stress, they substantially reduce reliance on transpirational cooling and photochemical energy dissipation, thereby interacting synergistically with NPQ and stomatal regulation.

### 4.1. Phytohormonal Regulation of Heat Stress Responses

Abscisic acid (ABA) enhances heat resistance by stimulating antioxidant enzyme activity, reducing lipid peroxidation, and improving cell viability under stress [94]. At the same time, ABA promotes stomatal closure, which limits transpirational cooling and indirectly increases Foliar temperature, illustrating a fundamental trade-off between water conservation and thermal regulation. As emphasized in the second review, ABA functions as a central integrator of drought–heat stress cross-talk, coordinating hydraulic status with cellular protection mechanisms. Heat stress increases auxin concentration, which supports thermotolerance by reducing oxidative damage and maintaining antioxidant enzyme levels [95]. Application of auxin analogs alleviates heat-induced reproductive impairments, including pollen sterility, by stimulating endogenous auxin production and ROS signaling in pistils [96]. Brassinosteroids (BRs) enhance thermotolerance by supporting carbon assimilation, modulating ROS metabolism, stabilizing translational machinery, and minimizing cellular damage under high temperatures [97,98]. These effects collectively contribute to maintaining photosynthetic performance during thermal stress, a point strongly emphasized in the comparative analyses discussed in the second review.

Gibberellins (GAs) influence heat responses by regulating salicylic acid (SA) synthesis and modulating phytohormone signaling through PHYTOCHROME-INTERACTING FACTORS (PIFs), thereby linking thermomorphogenesis with stress adaptation. Ethylene affects antioxidant defenses and interacts with jasmonates (JA) under heat stress, although it can suppress thermotolerance in some contexts, as demonstrated in the *ein2* mutant [99,100,101]. Salicylic acid enhances heat tolerance by improving photosynthesis and promoting proline accumulation, acting synergistically with ABA and ethylene pathways [102,103]. Together, ABA, auxin, BRs, GAs, ethylene, and SA regulate ROS metabolism, antioxidant defenses, cell division, and reproductive development, enabling plants to maintain growth and reproductive success under heat stress [104,105].

### 4.2. Xanthophyll Cycle Pigments and Regulated Thermal Energy Dissipation

Beyond hormonal regulation, the dissipation of excess excitation energy within chloroplasts represents a critical photoprotective mechanism during heat stress. Xanthophyll cycle pigments provide a regulated biochemical pathway for converting absorbed light energy into heat, thereby protecting the photosynthetic apparatus from photodamage under conditions where photochemical energy utilization is constrained. The reversible conversion of violaxanthin to zeaxanthin via antheraxanthin, catalyzed by violaxanthin de-epoxidase under acidic thylakoid lumen conditions, enhances non-photochemical quenching (NPQ) capacity and allows excess absorbed excitation energy to be safely dissipated as heat [106,107]. This process is closely linked to the formation of the proton motive force across the thylakoid membrane and is therefore sensitive to both light intensity and temperature. Under heat stress, increased membrane fluidity and altered electron transport dynamics can enhance lumen acidification, thereby promoting xanthophyll cycle engagement and rapid NPQ induction. Importantly, NPQ does not generate new thermal energy; instead, it regulates the timing and localization of heat release within the chloroplast, preventing the formation of reactive triplet chlorophyll states and singlet oxygen that would otherwise exacerbate oxidative damage. Notably, abscisic acid (ABA) biosynthesis shares metabolic precursors with xanthophyll pigments, establishing a biochemical link between drought signaling, photoprotection, and regulated AEE dissipation as heat [108,109]. This shared metabolic origin enables coordination between stomatal regulation and chloroplast photoprotection during combined heat and water stress. Under such conditions, reduced transpirational cooling increases Foliar temperature, while enhanced NPQ limits photoinhibition, illustrating how hormonal and pigment-based mechanisms jointly shape foliar thermal balance [106,107,109]. However, it is essential to distinguish regulated NPQ-mediated AEE dissipation as heat from total foliar heat emission. Only a fraction of absorbed light energy is dissipated through NPQ; the majority of energy not used for photochemistry is ultimately released as heat through unavoidable thermodynamic processes associated with excitation relaxation, basal metabolic activity, and inefficiencies in photosystems I and II [110]. Consequently, NPQ primarily modulates photoprotection and energy partitioning rather than serving as the dominant source of leaf-derived heat. This distinction is crucial for accurately interpreting the contribution of photochemical regulation to a plant’s energy balance and for avoiding overestimation of NPQ-driven thermal effects at the canopy or ecosystem scale [40].

### 4.3. Calcium, ROS, and Heat Shock Signaling Networks

Thermotolerance further depends on complex signaling cascades that translate thermal perception into cellular responses. A rapid cytosolic Ca^2+^ influx is one of the earliest cellular responses to heat, activating Ca^2+^ sensors such as calmodulin (CaM) [111]. In *Arabidopsis*, overexpression of *AtCaM3* enhances survival under heat shock through interactions with calmodulin-binding protein kinase 3 (CBK3) and protein phosphatase 7 (PP7), which regulate HSFA1 activity [112,113]. In rice, heat-induced cytosolic Ca^2+^ elevation and OsCaM1-1 upregulation activate downstream genes encoding CBK3, PP7, HSFs, and HSPs, strengthening thermotolerance [114]. Ca^2+^ signaling intersects closely with ROS networks; for instance, overexpression of annexin OsANN1 enhances SOD and CAT activities, helping to maintain cellular redox balance under heat stress [113]. Heat stress induces the production of ROS such as H_2_O_2_, O_2_^−^, ^1^O_2_, and OH^−^, which can damage membranes, proteins, and nucleic acids [105]. Plants counter these effects through antioxidant enzymes, including SOD, CAT, and APX [82]. Antioxidant capacity is often enhanced under heat stress in species such as wheat, tomato and radish, although severe or prolonged stress can overwhelm these defenses [115,116].

### 4.4. Transcriptional Control of Thermotolerance

Thermotolerance is ultimately consolidated through transcriptional reprogramming that coordinates cellular protection, repair, and metabolic adjustment under elevated temperatures [117,118]. Heat shock proteins (HSPs) are central components of this response, functioning as molecular chaperones that prevent protein denaturation, aggregation, and loss of enzymatic activity during heat stress [117,119]. By stabilizing photosynthetic complexes, metabolic enzymes, and membrane-associated proteins, HSPs help preserve cellular integrity and sustain physiological function under thermal stress [117,118]. Small heat shock proteins (sHSPs) play a particularly important role during the early phases of heat stress by associating with membranes and photosynthetic complexes, thereby stabilizing lipid bilayers and preventing irreversible damage [120]. Enhanced accumulation of sHSPs has been correlated with increased thermotolerance in crops such as rice, indicating their importance in maintaining photosynthetic efficiency and membrane stability under high temperatures [121]. In addition to their chaperone activity, several HSPs can perceive changes in cellular redox status and respond to reactive oxygen species (ROS), thereby integrating heat and oxidative stress signaling pathways [122]. Heat shock transcription factors (HSFs) orchestrate the heat shock response by activating genes encoding HSPs, antioxidant enzymes, and metabolic regulators [118]. In *Arabidopsis*, exposure to elevated temperatures induces the expression of a large proportion of heat-responsive genes, including *HSFA2*, *HSFBs*, *DREB2A*, and *MBF1c* [112,118]. Among these, *HSFA2* plays a key role in acquired thermotolerance by sustaining HSP expression during prolonged or repeated heat stress, and its overexpression enhances plant survival under high temperatures [113]. Members of the *HSFA1* family act as primary regulators of the heat shock response by integrating upstream signals such as Ca^2+^ influx and ROS accumulation with transcriptional activation of heat-responsive genes [113,123]. Beyond the classical heat shock pathway, additional transcription factor families, including WRKY, NAC, MYB, bHLH, and AP2/ERF, modulate thermotolerance by regulating stomatal behavior, antioxidant capacity, chloroplast function, and stress-responsive metabolism [124]. These transcription factors are frequently activated through MAPK-dependent phosphorylation cascades and operate at the intersection of hormonal signaling, calcium dynamics, and redox regulation [124]. Secondary messengers such as Ca^2+^, ROS, and nitric oxide rapidly transmit heat stress signals, enabling tight temporal control of gene expression during stress perception and early response phases [111,125]. Heat stress also disrupts protein homeostasis, triggering the unfolded protein response (UPR) in both the endoplasmic reticulum and chloroplasts [126]. Transcription factors such as *HSFA1s*, NAC family members, and *JUB1* integrate UPR signaling with oxidative stress responses, promoting cellular repair, metabolic adjustment, and survival under prolonged thermal stress [127,128]. Through these multilayered transcriptional networks, plants transition from short-term heat avoidance and protection toward longer-term acclimation, ensuring sustained growth, reproductive success, and resilience in warming environments [118,128]. An overview of the major regulators and mechanisms contributing to plant thermotolerance and foliar thermal balance, including passive structural traits, phytohormones, transcription factors, signaling pathways, and protective systems, is summarized in Table 1.

## 5. Conclusions

Absorption of energy in excess (AEE) exposes plants to several interacting forms of stress, including heat stress, photooxidative stress, photoinhibition, photorespiration, and osmotic stress. These stresses rarely occur in isolation. Instead, they are tightly linked through the regulation of photochemical energy use, non-photochemical quenching (NPQ), stomatal conductance, and associated signaling pathways, all of which together shape plant performance under adverse environmental conditions. This review highlights that foliar temperature regulation during AEE is not controlled by a single mechanism but emerges from how AEE is distributed and managed within the photosystem reaction centers and then within the leaf. When warming and drought restrict stomatal conductance, transpirational cooling becomes less effective, shifting the balance toward increased reliance on photochemical to non-photochemical and photorespiratory processes, which may induce cell death [49,83,84]. Under such conditions, foliar heat stress becomes more likely, whereas alterations in photochemical electron transport or regulated energy dissipation can, in some cases, modify short-term leaf temperature dynamics without causing irreversible cellular damage. The primary aim of this review is to integrate these well-established processes into a unified, coherent framework that links leaf-level energy partitioning with environmental constraints imposed by global warming. While current climate and vegetation models successfully capture canopy energy exchange and transpiration, leaf-internal energy redistribution associated with AEE is often simplified or treated implicitly. Explicit consideration of these interactions may help refine predictions of plant heat stress responses and vegetation–climate feedbacks under future warming scenarios [42,67].

## Figures and Tables

**Table 1:** Established regulators and mechanisms contributing to plant thermotolerance and foliar thermal balance.

Category	Regulator	Molecular/Structural Type	Primary Function Under Heat Stress	Mechanistic Basis	References
Passive structural traits	Leaf size, shape, orientation; cuticle, trichomes	Morphological traits	Heat avoidance; ↓ foliar temperature	Reduced radiation absorption, thinner boundary layer, enhanced convective and radiative heat loss	[47,91,92]
	Canopy architecture, self-shading	Whole-plant traits	Buffer thermal extremes	Reduced solar interception; canopy-scale thermal heterogeneity	[34,129]
Phytohormone	Abscisic acid (ABA)	Sesquiterpene hormone	↑ Thermotolerance ↓ transpiration	Ca^2+^ influx, antioxidant enzyme induction (SOD, CAT, APX), membrane stabilization; stomatal closure	[130,131,132]
	Auxins (IAA, NAA)	Indole hormones	Developmental heat adaptation	Maintains pollen viability, cell division, antioxidant balance	[133,134,135]
	Cytokinins (CKs)	Adenine derivatives	Yield stability under heat	Delay senescence, maintain grain filling and sink–source relations	[3,136,137]
	Brassinosteroids (BRs)	Steroid hormones	↑ Thermotolerance	Enhance photosynthesis, ROS detoxification, translational stability	[138,139,140]
	Gibberellins (GAs)	Diterpenoid hormones	Developmental plasticity	GA–PIF4–auxin integration; flowering and thermomorphogenesis	[135,141,142]
	Ethylene	Gaseous hormone	Context-dependent	Modulates oxidative stress and senescence; EIN2 may negatively regulate thermotolerance	[102,117,135]
	Salicylic acid (SA)	Phenolic hormone	Basal and acquired thermotolerance	Enhances antioxidant capacity, proline accumulation, pollen protection	[102,143,144]
	Jasmonic acid (JA)	Lipid hormone	Basal thermotolerance	Activates stress-responsive transcriptional networks	[101,145]
Transcription factor	HSFA1 (A/B/D/E)	HSF family	Master regulator of HSR	Activates HSP genes and stress-related enzymes	[117,118]
	HSFA2	HSF family	Acquired thermotolerance	Sustains HSP expression during prolonged or repeated stress	[113]
	HSFBs	HSF family	HSR fine-tuning	Modulate intensity and duration of heat response	[128]
	DREB2A	AP2/ERF family	Heat and drought response	Activates abiotic stress-responsive genes	[113,146]
	MBF1c	Transcriptional co-activator	ROS-linked thermotolerance	Bridges oxidative signaling and HSR	[147]
	NAC TFs (e.g., NAC019, ONAC066)	NAC family	Heat and oxidative stress tolerance	Direct activation of HSFs and stress-responsive genes	[127,128]
	WRKY TFs	WRKY family	Heat–ROS signaling	MAPK-mediated regulation of stress genes	[124]
	PIF4	bHLH family	Thermomorphogenesis	Integrates GA, auxin, and temperature signaling	[133,135]
Signaling system	Ca^2+^–Calmodulin (CaM3)	Ca^2+^ sensor	Heat signal transduction	Activates HSFs via CBK3 and PP7	[111,113]
	ROS signaling	Redox system	Stress perception and signaling	H_2_O_2_ activates HSR and antioxidant genes	[125]
Protective system	Heat Shock Proteins (HSPs)	Molecular chaperones	Protein protection	Prevent denaturation and aggregation	[119,121]
	Antioxidant enzymes (SOD, CAT, APX)	Enzymatic defense	ROS detoxification	Prevent lipid peroxidation and membrane damage	[148,149]

**Notes:** ↑ and ↓ indicate an increase and a decrease, respectively.

## Data Availability

No new data were created or analyzed in this study. Data sharing is not applicable to this article.
